# Propofol Induces the Expression of Nrf2 and HO-1 in *Echinococcus granulosus* via the JNK and p38 Pathway In Vitro

**DOI:** 10.3390/tropicalmed8060306

**Published:** 2023-06-03

**Authors:** Guangyi Luo, Bin Ma, Yufeng Jiang, Hailong Lv

**Affiliations:** 1 Affiliated Hospital of Southwest Jiaotong University, The Third People’s Hospital of Chengdu, Chengdu 610031, China; 2 Section for Hepatopancreatobiliary Surgery, Department of General Surgery, Affiliated Hospital of Southwest Jiaotong University, The Third People’s Hospital of Chengdu, Chengdu 610031, China; 3 Department of General Surgery, Jinxiang People’s Hospital, Jining 272200, China; 4 School of Basic Medicine, Chengdu Medical College, Chengdu 610500, China

**Keywords:** *Echinococcus granulosus*, protoscoleces, MAPK, Nrf2, propofol

## Abstract

The purpose of this study was to establish the relationship between mitogen-activated protein kinase (MAPK) and Nrf2 signaling pathways in *Echinococcus granulosus* (*E. granulosus*). *E. granulosus* protoscoleces (PSCs) cultured in vitro were divided into different groups: a control group, PSCs were pretreated with various concentrations of propofol followed by exposure to hydrogen peroxide (H_2_O_2_), and PSCs were pretreated with MAPK inhibitors, then co-treated with propofol and incubated in the presence of H_2_O_2_. PSCs activity was observed under an inverted microscope and survival rate was calculated. Reactive oxygen species (ROS) was detected by fluorescence microscopy, western blotting was used to detect the expression of Nrf2, Bcl-2, and heme oxygenase 1 (HO-1) in the PSCs among different groups. Pretreatment of PSCs with 0–1 mM propofol for 8 h prevented PSCs death after exposure to 0.5 mM H_2_O_2_. PSCs were pretreated with PD98059, SB202190, or SP600125 for 2 h, co-treated with propofol for an additional 8 h, and then exposed to 0.5 mM H_2_O_2_ for 6 h. On day 6, the PSCs viability was 42% and 39% in the p38 and JNK inhibitor groups, respectively. Additionally, pretreatment with propofol significantly attenuated the generation of ROS following H_2_O_2_ treatment. Propofol increased the expression of Nrf2, HO-1, and BCL2 compared with that of the control group. Pretreatment PSCs with SP600125 or SB202190, co-incubation with propofol and H_2_O_2_, can reduce the expression of Nrf2, HO-1, and BCL2 (*p* < 0.05). These results suggest that propofol induces an upregulated expression of HO-1 and Nrf2 by activation of the JNK and p38 MAPK signaling pathways. This study highlights the cross role of metabolic regulation of ROS signaling and targeting signalling pathways that may provide a promising strategy for the treatment of *E. granulosus* disease.

## 1. Introduction

Cystic echinococcosis (CE), caused by the metacestode of *Echinococcus granulosus* (*E. granulosus*), affects both humans and animals. It is a global public health and economic problem, especially in Central Asia, Mediterranean countries, Northern Africa, and South America [1]. Currently, CE treatment varies mainly according to the cyst type classification, size, location, and complications, as well as available medical expertise and equipment [2]. Curative treatment is achieved by the complete removal of the cyst, regardless of location [3]. However, the post-operative recurrence of CE due to cyst rupture or spilling of cyst contents (protoscoleces, PSCs) during surgery is a major surgical complication of CE. Thus, the use of effective scolicidal agents during the procedure is an essential part of the surgical technique and helps lower the risk of spilling viable PSCs. So far, there are some agents that are routinely used in open CE cysts (such as hypertonic saline, silver nitrate, ethanol, mannitol, chlorhexidine gluconate, etc.) [4,5] and others that may be used experimentally (Berberis vulgaris [6], selenium nanoparticles [7], 5-hydroxyl-1,4-naphthoquinone, etc.) [8]. Unfortunately, the consumption of these agents has been limited because of their low efficacy, toxicity, and severe side effects such as sclerosing cholangitis and liver necrosis. Therefore, it is important to find new scolicidal agents with fewer side effects, low cost, and higher efficacy, as there is an urgent need for surgeons.

An unexplored issue associated with the parasite’s persistence in its host is how the parasite can survive the oxidative stress resulting from parasite endogenous metabolism and host defenses. During the early developmental stages of echinococcosis, both cysts and hosts produce large amounts of unstable reactive oxygen species (ROS) that trigger oxidative stress responses, damage proteins, membrane lipids, and DNA, leading to cell and tissue death. Despite this hostile host environment, PSCs are able to survive and differentiate into secondary hydatid cysts, relying for that on the production of antioxidant molecules and enzymes that prevent oxidative damage to macromolecules [9], and antioxidant enzymes play a crucial role in protecting parasites against host metabolism and oxidative damage [10].

The transcription factor Nrf2 is best known as one of the main orchestrators of the cellular xenobiotic and oxidative stress response. It regulates the cellular defense against toxic and oxidative insults through the expression of genes involved in oxidative stress response and drug detoxification. In addition to antioxidant responses, Nrf2 is involved in many other cellular processes, including metabolism and inflammation, and its functions are beyond the originally envisioned. Under normal conditions, Nrf2 is bound to Kelch-like ECH-associated protein 1 (Keap1) in the cytoplasm, and binding to Keap1 targets Nrf2 for proteasomal degradation to maintain low intracellular levels. In response to oxidative stress, Keap1 is inactivated, causing the release of active, stabilized Nrf2 that translocates to the nucleus to activate transcription of a variety of antioxidant and detoxification genes [11,12]. The majorities of excessive ROS are scavenged by Nrf2 and its downstream target proteins and can effectively resist apoptosis caused by oxidative damage [13].

Mitogen-activated protein kinase (MAPK) is a group of protein kinases that play an essential role in signal transduction by modulating gene transcription in the nucleus in response to changes in the cellular environment. MAPK pathways are regulated by growth factors, mitogens, hormones, and stress stimuli and transmit a signal from the cell surface to the nucleus. MAPK controls different biological responses such as cell growth, proliferation, differentiation, and cell death [14]. The three groups of MAPK that have been well characterized include the extracellular signal-regulated kinase (ERK), the c-Jun N-terminal kinase (JNK), and the p38 MAPK. 

There is a complex interaction between MAPK and the Nrf2/HO-1 signaling pathway, which is inconsistent under different conditions of research. Different MAPK can affect the activation of Nrf2 and thus affect the expression of heme oxygenase 1 (HO-1). Previous studies [15] have found, by analyzing amino acid sequences, that the interaction between MAPK and Nrf2 may be related to the presence of MAPK protein phosphorylation sites in the reverse activation domain of Nrf2. The possible mechanism by which p38 promotes Nrf2/HO-1 activation and expression is that p38 phosphorylates Nrf2 and separates it from Keap1 in the cytosol, thereby promoting the activation of Nrf2 [16,17]. In addition, the regulation may also be related to p38 promoting the nuclear transfer of related proteins and protecting Nrf2 from degradation [18]. Studies have shown that p38, ERK1/2, and JNK can synergistically activate the Nrf2/HO-1 pathway, and the application of inhibitors, respectively, can significantly inhibit Nrf2 nuclear transfer and reduce the expression level of HO-1. Nrf2, in turn, can affect JNK activation by regulating gene expression [19,20].

Propofol (2,6-diisopropylphenol) has been widely used for inducing and maintaining anesthesia. Apart from its anesthetic advantages, propofol has been reported to have many other effects such as antioxidative and anti-inflammatory properties [21,22]. Gu et al. [23] found that propofol-induced protection of human neuroblastoma cells (*SH-SY5Y*) against H_2_O_2_ is associated with HO-1 via the ERK Pathway. Our previous study has confirmed that MAPK and Nrf2 are both expressed in *E. granulosus* PSCs and play an important role in the growth and development of *E. granulosus* [24,25]. Under the stimulation of other injurious effects of oxidative stress, the interaction between MAPK and Nrf2/HO-1 signaling pathways in *E. granulosus* has not been established experimentally. In this study, H_2_O_2_ and propofol were used to culture *E. granulosus* PSCs in vitro, and the expression of Nrf2 and HO-1 protein was detected. Then we made the MAPK inhibitors, preliminarily to explore the relationship between MAPK and Nrf2 signaling pathways in the *E. granulosus* PSCs. 

## 2. Materials and Methods

### 2.1. Drug Assays

HO-1 rabbit polyclonal antibody, Nrf2 rabbit polyclonal antibody, and B-cell leukemia/lymphoma-2 (BCL-2) antibody were purchased from Sigma. Non-ATP-competitive MEK inhibitors (PD98059), selective p38 MAPK inhibitors (SB202190), and JNK inhibitors (SP600125) were purchased from Jiancheng (Nanjing, China) and were dissolved in dimethyl sulphoxide (DMSO) at a drug concentration of 0.5 M. A HO-1 and ROS detection kit was obtained from Jiancheng (Nanjing, China). Propofol was purchased from Xi’an Libang Enterprises (Xi’an, China).

### 2.2. Parasite Sample Collection and Culture

PSCs of *E. granulosus* were aseptically isolated from liver hydatid cysts obtained from infected sheep scheduled for routine slaughter in Shihezi, West China. PSCs cultures were performed as previously described [22]. RPMI1640 medium was utilized for the culture of PSCs, supplementing with 10% fetal bovine serum (FBS), 100 U/mL penicillin, and 100 μg/mL streptomycin at 37 °C in a humidified 5% CO_2_ incubator. 

### 2.3. Evaluation of Drug Treatment on PSCs of E. granulosus 

About 300 PSCs were seeded onto each well of the 96-well plates by dividing into the following groups: first, PSCs were pretreated with various concentrations (0–1 mM) of propofol for 24 h. Second, PSCs were pretreated with various concentrations (0–1 mM) of propofol for 8 h followed by exposure to 0.5 mM of H_2_O_2_ for 6 h. Third, PSCs were pretreated with PD98059 (an inhibitor of the ERK), SB202190 (an inhibitor of p38), and SP600125 (an inhibitor of JNK) for 2 h, co-treated with 1 mM propofol for another 8 h, and then PSCs were incubated for 6 h in the presence of H_2_O_2_. PSCs were collected to evaluate the viability via the eosin dye exclusion test (1 g of eosin powder in 1000 mL distilled water) followed by microscopically. 

### 2.4. ROS Levels Was Detected by Fluorescence Microscopy

*E. granulosus* PSCs were cultured as mentioned above. ROS accumulation was detected by fluorescence microscopy using DCFH-DA as the protocol reported previously [22]. The PSCs were observed under a fluorescence microscope according to the manufacturer’s instructions.

### 2.5. Examinations about HO-1 Activity Assay

Total PSCs proteins were extracted, and the activity of HO-1 was measured using HO-1 ELISA according to the manufacturer’s instructions. Protein extracts from PSCs were treated with chemical reagents according to a previous description, and then the reaction buffer with HO-1 ELISA kit was added into these proteins following the instructions. The mixture was placed into 96-well plates and incubated for 1 h at 37 °C, and we used a microplate plate reader (Bio-RAD, Hercules, CA, USA) to measure the absorbance of ρNA at 450 nm. By subtracting the absorbance of negative controls from specific values, we can calculate the mean values of triplicate measurements.

### 2.6. Western Blotting

The Western blot (WB) analysis was performed as previously described [22]. The primary antibodies used in this study were: BCL2, Nrf2, HO-1 (1:1000 dilution) from (Santa Cruz Biotechnology, Santa Cruz, CA, USA), and β-actin (1:5000 dilution) from (Sigma–Aldrich, St. Louis, MO, USA). Secondary antibodies were goat anti-rabbit IgG.

### 2.7. Statistical Analyses 

The data are given as the means ± SD. Data were analyzed via one-way ANOVA followed by Student’s *t*-test using SPSS 20.0 software. WB grayscale was analyzed using Image J V1.8.0 Software and plotted using Prism 9.0 (GraphPad Software, San Diego, CA, USA). Statistical significance was defined as a *p* value < 0.05.

## 3. Results

### 3.1. In Vitro Effects of H_2_O_2_ and Propofol on E. granulosus PSCs Viability

**Figure 1:**
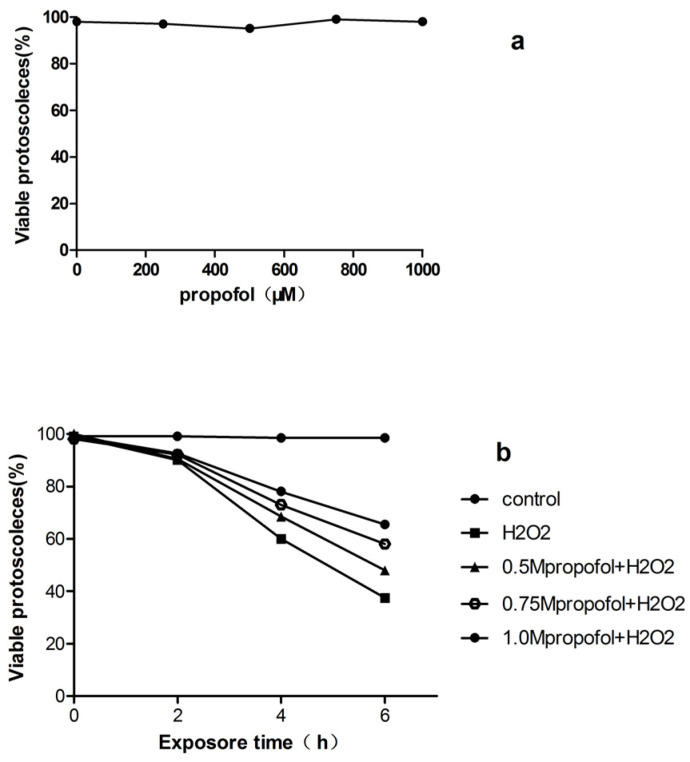
Viability of protoscoleces incubated in vitro with propofol and H_2_O_2_. (**a**) PSCs were exposed to various concentrations of propofol for 24 h; (**b**) PSCs were treated with propofol (0.5–1 mM) for 8 h then incubated with H_2_O_2_ (0.5 mM) for a further 6 h, PSCs viability was measured by 0.1% eosin staining under light microscope. Results are representative of three independent experiments. PSCs, Protoscoleces; H_2_O_2_.

First, the cytotoxic effects of propofol on PSCs after 24 h of exposure were examined. The relative survival rate of PSCs treated with 0–1 mM propofol was over 95%, as shown in Figure 1a, indicating that propofol at these doses does not contribute significantly to cytotoxicity in PSCs. Then, the effect of propofol on H_2_O_2_-induced PSCs death was evaluated. PSCs were treated with propofol (0.5–1 mM) for 8 h, after which, the PSCs were exposed to 0.5 mM H_2_O_2_ in fresh medium for 6 h. As shown in Figure 1b, the survival rates of PSCs treated with 0.5 mM, 0.75 mM, and 1 mM propofol were 48%, 58%, and 65.5% at day 6, respectively. The PSCs viability was only 38% at day 6 in the H_2_O_2_ group.

**Figure 2:**
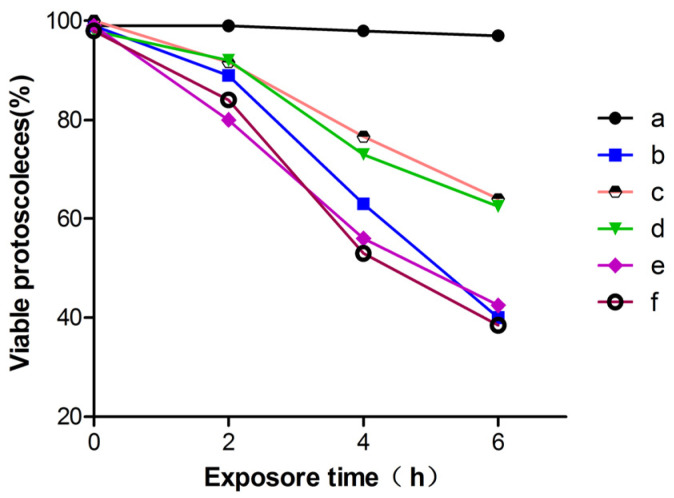
Viability of protoscoleces incubated in vitro with inhibitors of ERK, p38, and JNK. (a) control group; (b) PSCs were treated with H_2_O_2_ (0.5 mM) for 6 h; (c) PSCs were treated with 1 mM propofol for 8 h, after that, the PSCs was exposed to 0.5 mM H_2_O_2_ in fresh medium for another 6 h; (d–f) PD98059, SB202190, and SP600125, respectively, were used in pretreatment of PSCs for 2 h, and then PSCs were co-treated with 1 mM propofol for another 8 h, after that, the PSCs were exposed to 0.5 mM H_2_O_2_ in fresh medium for 6 h, PSCs viability was measured by 0.1% eosin staining under light microscope. Results are representative of three independent experiments. MAPK, mitogen-activated protein kinases; ERK, extracellular signal-regulated kinases; JNK, C-Jun N-terminal kinases.

The viability of protoscoleces incubated in vitro with inhibitors of ERK, p38, and JNK were shown in Figure 2. On day 6, the PSCs viability was 42% and 39% in the p38 and JNK inhibitor groups (e and f group), respectively, which was close to the group of H_2_O_2_. The activity of the ERK inhibitor group (d group) was close to that of the propofol group (c group), it seems that the ERK inhibitor group failed to block the protective effect of propofol on oxidative stress-induced death. Taken together, these results indicate that propofol inhibits H_2_O_2_-induced death in PSCs, and the p38 and JNK signaling pathways participate in this process.

### 3.2. Effects of Propofol on H_2_O_2_-Induced BCL2 Expression in E. granulosus PSCs

**Figure 3:**
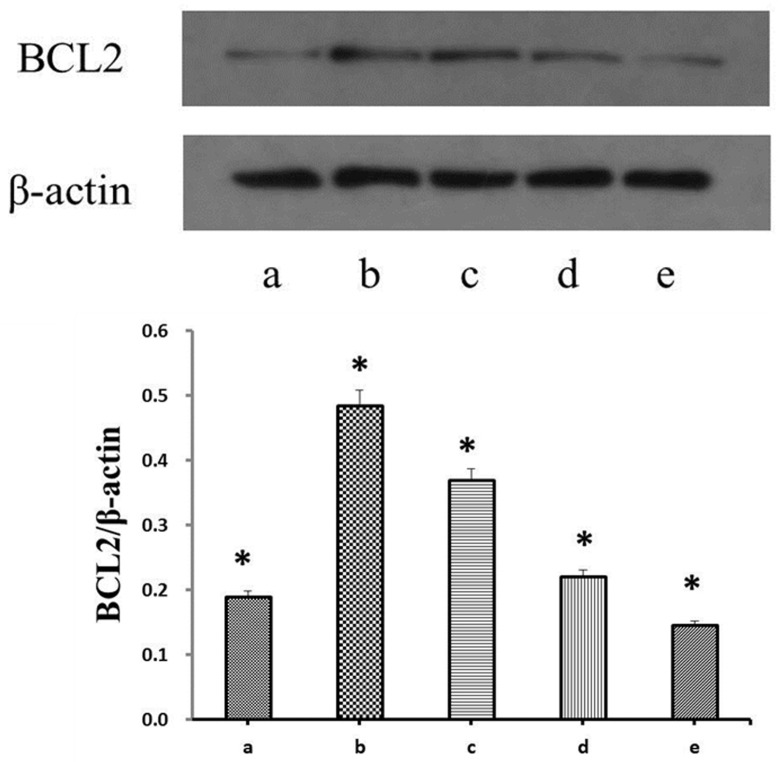
The expression of BCL2 after incubation with different drugs. (a) PSCs were treated with H_2_O_2_ (0.5 mM) for 6 h; (b) PSCs were treated with 1 mM propofol for 8 h and then incubated with 0.5 mM H_2_O_2_ for 6 h; (c–e) PD98059, SB202190, or SP600125, respectively, were used in pretreatment of PSCs for 2 h, and then PSCs were co-treated with 1 mM propofol for another 8 h, after that, the PSCs were exposed to 0.5 mM H_2_O_2_ for 6 h. BCL2, B-cell lymphoma 2. BCL2 protein expression in each group was indicated by BCL2/β-actin. Bar graph indicates: * compared with the control group, *p* < 0.05.

The expression of BCL2 was evaluated to determine the activation of H_2_O_2_-induced apoptosis in PSCs. Western blotting showed that BCL2 expression was markedly reduced in the H_2_O_2_-treated PSCs compared to the control group (*p* < 0.05). However, propofol pretreatment attenuated the down-regulated expression of BCL2 that was induced by H_2_O_2_ (*p* < 0.05). In agreement with the results of viability, there was a significant reduction in BCL2 activity in the SB202190 and SP600125 pretreatment of PSCs, which were also co-treated with propofol, then treated with H_2_O_2_ (Figure 3). 

### 3.3. Effects of H_2_O_2_, Propofol and MAPK Inhibitors on ROS in E. granulosus PSCs In Vitro

**Figure 4:**
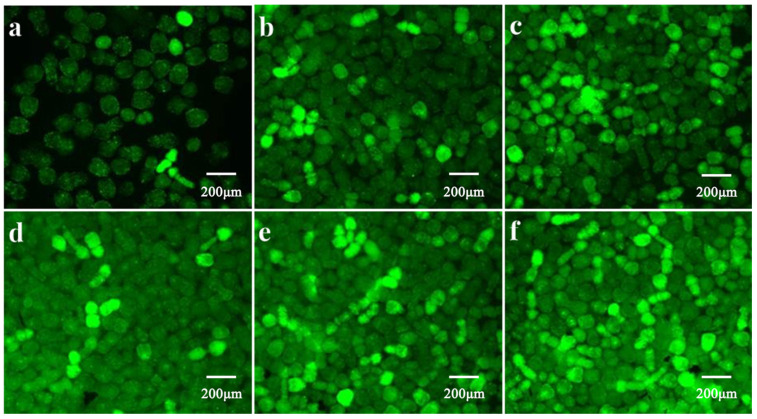
ROS fluorescence intensity of protoscoleces after incubation with different drugs. (**a**) control group; (**b**) PSCs incubated with propofol (1 mM) for 8 h and then incubated with 0.5 mM H_2_O_2_ for 6 h; (**c**) PSCs were treated with H_2_O_2_ (0.5 mM) for 6 h; (**d**–**f**) PD98059, SB202190, or SP600125, respectively, were used in pretreatment of PSCs for 2 h, and then PSCs were co-treated with 1 mM propofol for another 8 h, after that, the PSCs were exposed to 0.5 mM H_2_O_2_ for 6 h. Results are representative of three independent experiments. ROS, Reactive oxygen species; DCFH-DA: 2′,7′-dichlorofluorescin diacetate.

To determine whether the JNK and p38 MAPK signaling pathways participate in the propofol-induced inhibition of ROS production in PSCs of *E. granulosus*, the PSCs were treated with a DCFH-DA probe (Figure 4). The results show that treatment with propofol significantly reduced H_2_O_2_-induced ROS generation, but co-incubation of the PSCs with propofol, H_2_O_2_, and SB202190 or SP600125 increased PSCs ROS production, indicating that JNK or p38 inhibitors attenuate the effect of propofol in PSCs. 

### 3.4. MAPK Signaling Pathways Participate in Propofol-induced Nrf2 and HO-1 Expression

**Figure 5:**
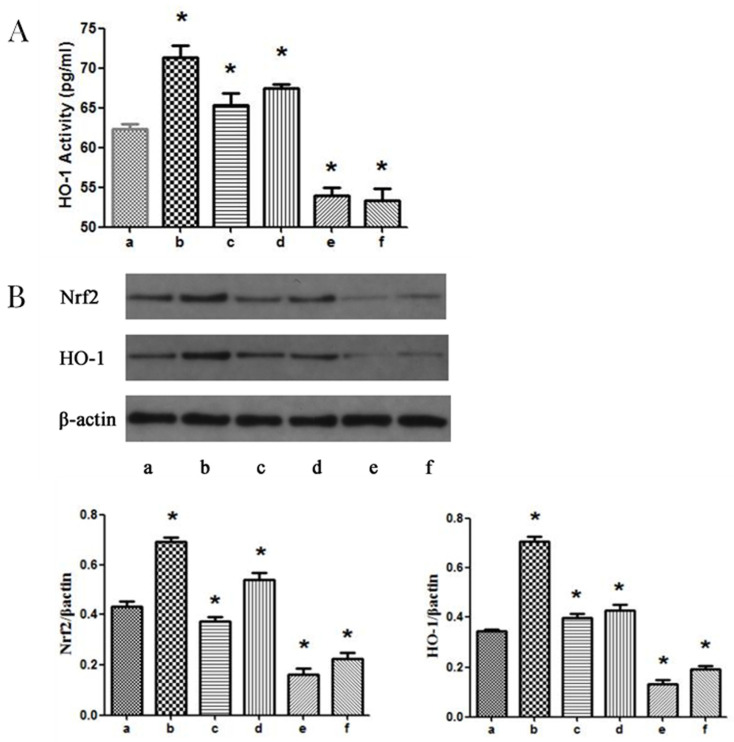
(**A**): Expression of HO-1 between different group detected by HO-1 ELISA kit. (**B**): Expression of Nrf2 and HO-1 between different groups detected by western blot. (a) control group; (b) PSCs were treated with 1 mM propofol for 8 h and then incubated with 0.5 mM H_2_O_2_ for 6 h; (c) PSCs were treated with H_2_O_2_ (0.5 mM) for 6 h; (d–f) PD98059, SB202190, or SP600125, respectively, were used in pretreatment of PSCs for 2 h, and then PSCs were co-treated with 1 mM propofol for another 8 h, after that, the PSCs were exposed to 0.5 mM H_2_O_2_ for 6 h. Bar graph indicates: * compared with the control group, *p* < 0.05. Results are representative of three independent experiments. Nrf2, nuclear factor erythroid-2-related factor 2; ARE, antioxidant components; HO-1, hemeoxygenase-1; ELISA, enzyme-linked immunosorbent assay.

To analyze the mechanism by which these pathways could participate, propofol-induced Nrf2 and HO-1 expression were examined. Using a colorimetric HO-1 assay kit, it was found that the JNK and p38 MAPK signaling pathways participate in propofol-induced HO-1 expression (Figure 5A). The results indicate that propofol increased the expression of Nrf2 and HO-1 compared with that of the control group. Co-incubation of the PSCs with propofol, H_2_O_2_, and SP600125 or SB202190 reduced the expression of Nrf2 and HO-1, indicating that JNK and p38 MAPK are implicated in the expression of Nrf2 and HO-1. Moreover, PD98059 did not block the expression of Nrf2 and HO-1 (Figure 5B). These results indicate that JNK and p38 MAPK participate in the expression of Nrf2 and HO-1 induced by propofol.

## 4. Discussion

CE remains a serious clinical problem worldwide. Currently, surgery is the primary treatment option for complicated cases of CE. However, the leakage of cyst contents after operation can lead to secondary dissemination or relapse. Many drugs used in CE treatment have been found to be toxic in some patients [3]. To date, a parasiticidal ideal agent that is both effective and riskless has not been identified. Our previous experiments confirmed that the use of MAPK or Nrf2 signaling pathway inhibitors can affect PSCs viability by denaturing and structurally disrupting the *E. granulosus* PSCs [24,25]. The purpose of this study was to elucidate the association between MAPK and Nrf2 signaling pathways in vitro in *E. granulosus*, which may be useful in providing areas for more drug research in the treatment of CE. 

Recent research suggests that MAPK and Keap1-Nrf2-ARE signaling pathways are involved in the pathological process of inflammation and oxidative stress [18,26,27]; it is suggested that MAPK has an effect on Nrf2 activity. Under oxidative stress or other injurious stimuli, cytokines can activate MAPK molecules and promote Nrf2 transfer, thereby upregulating the expression of HO-1. Endogenous carbon monoxide (CO), mainly produced by the degradation of heme by heme oxygenase (HO), plays an important role in the regulation of various MAPKs [28]. However, the role of these molecules is not completely consistent under different stimulating factors and in different tissue cells.

Low concentrations of H_2_O_2_ can induce an increase in ROS production [29]. We chose a low concentration of H_2_O_2_ at 0.5 nM to induce ROS generation on the *E. granulosus* PSCs. There was no difference in the activity of PSCs culture with H_2_O_2_ for 6 h and 24 h. In our study, the concentration and time of propofol used were selected by our previous experiments. In the concentration range of propofol 0.25 mM to 1 mM, it can be seen that the survival rate of PSCs increases as the concentration increases. However, compared with 1 mM, the concentration of propofol increased to 2 or 3 mM caused no difference in PSCs viability. 

Propofol was previously observed to be able to protect some cells against oxidant stress [22,23]. Our experimental results are consistent with this observation. The cytotoxic effects of propofol on PSCs exposure were also examined. The relative survival rate of PSCs treated with different concentration propofol was over 95%, indicating no toxicity stimulated by propofol. Our results show that the survival rates of PSCs treated with propofol and H_2_O_2_ were significantly increased compared with PSCs treated with H_2_O_2_. This protective effect may be related to the increased expression of Nrf2 and HO-1 induced by propofol. Most importantly, there was a marked decrease in cell viability with JNK and p38 MAPK inhibitors combined with propofol than the propofol alone. These results suggest that MAPK affects the viability of *E. granulosus* PSCs by regulating Nrf2 activity.

The main function of antiapoptotic Bcl-2 proteins is to restrain proapoptotic BAX/BAK, thus preserving mitochondrial outer membrane integrity. The function was supported by the observation that decreased Bcl-2 expression leads to increased oxidative stress while Bcl-2 overexpression leads to reduced oxidative stress [30]. Our results showed the expression of Bcl-2 in the H_2_O_2_ group was lower than in control group. While the expression of Bcl-2 was increased in PSCs treated with propofol and H_2_O_2_. It indicates a directly protective effect of propofol against H_2_O_2_-induced PSCs apoptosis. This finding is probably consistent with that of Yoon et al. [31], who also found propofol had a protective effect on H_2_O_2_-induced COS-7 cell apoptosis. However, when we treated PSCs with propofol and H_2_O_2_, and incubated with MAPK inhibitors, we observed dramatically reduced Bcl-2 levels in the JNK and p38 MAPK inhibitors but observed no change in PSCs in the ERK MAPK inhibitors. These results suggest that the protective effect of propofol in H_2_O_2_-induced PSCs apoptosis was associated with JNK and p38 MAPK activation.

Overexpression of HO-1 can increase the oxidation resistance of the body and reduce oxidative damage [32]. Propofol has a protective effect against ROS-mediated cell injuries, including apoptosis and autophagy, through inhibiting the production of ROS [33,34]. The possible mechanism may be related to inducing HO-1 overexpression [19]. In our experiments, the antioxidant enzyme HO-1 was induced by propofol in a concentration-dependent manner, which was related to inhibition of ROS production. We can observe that high concentrations of propofol significantly inhibit the expression of ROS. The results indicate that high concentrations of propofol against H_2_O_2_ could be exerting a direct free radical scavenging effect. Propofol increases overexpression of Nrf2, and the expression of the Nrf2 downstream gene HO-1 showed the same trend. The underlying mechanisms of propofol-induced Nrf2 and HO-1 overexpression has been confirmed in many previous studies. However, this phenomenon has not been confirmed in *E. granulosus*.

A large amount of literature [35,36] has reported that MAPK has an effect on the activity of Nrf2, but there are contradictory results on the regulation of Nrf2 activity by ERK, JNK, and p38 in different cells. The reason may be that the regulation of Nrf2 by MAPK is indirect, the stability of Nrf2 protein is not changed by Nrf2 phosphorylation, and the direct phosphorylation of Nrf2 by MAPK has limited effects on regulating Nrf2 activity [37]. Gu et al. [23] reported that propofol-induced protection of *SH-SY5Y* cells against H_2_O_2_ is associated with HO-1 via the ERK pathway. These results suggest that different stimulating factors and responsive tissues may have different effects on the activation of different MAPKs. As reported above, we used ERK, JNK, and p38 MAPK signaling inhibitors independently in the pretreatment of PSCs. Results revealed that the antioxidant stress response to propofol was attenuated by the inhibition of JNK and p38 signaling. However, ERK inhibitors did not prevent propofol from protecting cells from oxidative stress-induced death. It is speculated that this may be related to propofol downregulating the function of ERK, but further research is needed to support this conclusion. In this study, the data provide strong evidence that propofol induces Nrf2 and HO-1 expression in vitro in *E. granulosus* via the p38 MAPK or JNK pathways. Based on our present results, MAPK influences the activity of Nrf2 in vitro in *E. granulosus.*

In summary, there is a complex interaction between MAPK and Nrf2/HO-1 signaling pathways, and this role is not consistent under different study conditions. Our research has found that both MAPK and Nrf2 pathway inhibitors had a killing effect on the *E. granulosus* PSCs. In this study, H_2_O_2_ can generate a large amount of ROS and induce PSCs death by oxidative stress. Meanwhile, propofol can reduce the production of ROS and maintain the activity of PSCs by increasing the expression of Nrf2/HO-1. Then we found that the protective effect from propofol-induced antioxidant effects was blocked after the administration of the MAPK pathway (p38, JNK) inhibitors, respectively, which resulted in a significant decrease in Nrf2 and HO-1 protein expression and activity in PSCs, but ERK inhibitors had no significant effect in this process. Our result shows that propofol induces Nrf2 and HO-1 expression in vitro in *E. granulosus* via the p38 MAPK or JNK pathways. The relationship between the two pathways has now been clarified, and these findings might provide more insight for the development of novel targeted drugs for the treatment of CE. However, the specific mechanism underling this process still needs to be further investigated.

## 5. Conclusions

In conclusion, H_2_O_2_ and MAPK inhibitors can inhibit the vitality and induce the production of ROS in PSCs. Propofol promotes the expression of Nrf2 and HO-1 in PSCs, and the expression of HO-1 and Nrf2 was significantly decreased in PSCs treated with p38 or JNK inhibitors. These results suggest that the JNK and p38 MAPK signaling pathways may regulate Nrf2 and HO-1 expression in *E. granulosus* PSCs. The correlation study between MAPK and Nrf2 signaling pathway on *E. granulosus* will provide more ideas for the development of clearer novel drugs for targeted therapy of *E. granulosus.*

## Data Availability

All data analyzed are included in this published article.
